# Rates of sustainment in the Universal Stages of Implementation Completion

**DOI:** 10.1186/s43058-021-00250-6

**Published:** 2022-01-05

**Authors:** Dylan Randall Wong, Holle Schaper, Lisa Saldana

**Affiliations:** Oregon Social Learning Center, Eugene, OR USA

**Keywords:** Sustainment, Competency, Stages of Implementation, SIC, Sustainment rate

## Abstract

**Background:**

Sustainment is a desirable outcome of implementation, but its precise definition remains unclear, contributing to the difficulty of identifying a generalized rate of sustainment. Several studies and reviews on the topic differ on both definition and levels of analysis. Furthermore, methodological limitations might have influenced the results, including the unknown quality with which some interventions were delivered. The Universal Stages of Implementation Completion (UniSIC) is a standardized measurement tool that tracks the implementation process and milestone completion across a wide range of real-world implementations—this provides a unique opportunity to identify a generalized rate of sustainment.

**Methods:**

UniSIC data was captured from the SIC website on 27 September 2020 and included data from all sites (*n* = 1778) that had been tracked to date. Data were restricted to sites that achieved competency in program delivery, and thus had a newly adopted program worthy of sustainment. Dates and indicator variables of implementation activities were combined to form two alternate definitions of sustainment: sustained (start-up) was achieved if sites continued to deliver services 2 years past their *program start-up date*; sustained (competent) was achieved if sites continued to deliver services 2 years past their *competence and/or certification date*. Of sites eligible for inclusion based on these definitions (*N* = 208), descriptive analyses were conducted to determine a rate of sustainment for all programs that successfully started a program. These definitions were also applied to a combined sample for a general rate of sustainment among all sites. Rates of competency among both a sample of sites that started up and a combined sample were also identified.

**Results:**

The rate of competence was 58.5% and the rate of sustained (start-up) was 37.1%, while the rate of sustained (competent) was 25.1%. The rates of competence and sustainment among the combined samples were far lower: 15.6% for competence, 6.8% for sustained (start-up), and 4.4% for sustained (competent).

**Conclusions:**

These identified rates of sustainment are accurate initial estimates of sustainment of community-based practices, or in general. Future research on rates of sustainment should carefully define measures of sustainment and be transparent about the real-world conditions on which analyses are centered.

Contributions to the literature

- Our study provides insight into the study of sustainment rates by leveraging a repository of data collected using an implementation process measurement tool (the Stages of Implementation Completion) to operationalize sustainment.
- We discuss relevant issues in defining and measuring sustainment and advocate for the benefits of measuring sustainment of real-world implementation in real time.
- The sustainment rates found provide a useful benchmark for purveyors, organizational leadership, policymakers, and researchers alike as they seek to improve upon sustainment of the programs they support.

## Background

### Sustainment

Recent implementation literature has emphasized the sustainment phase of implementation [1–3]. Sustainment is the desired outcome of implementation, typically involving the goal of maintenance of the practice within the service setting [4, 5]. While there remains no consensus on an exact definition of sustainment [6], a comprehensive and influential review by Moore and colleagues identified five key constructs important to the definition of sustainment of a healthcare innovation: “(1) [occurring] after a defined period of time, (2) the program, clinical intervention, and/or implementation strategies continue to be delivered and/or (3) individual behavior change (i.e., clinician, patient) is maintained; (4) the program and individual behavior change may evolve or adapt while (5) continuing to produce benefits for individuals/systems” (p. 5) [4]. Other constructs identified in a review by Nadalin Penno et al. include sustainment as a process and as a post-implementation stage [7]. Given its importance, Moullin et al. recommended that implementation efforts “begin with sustainment in mind” (p. 9) [8].

However, there is little research on sustainment [1, 2], which may be due to the challenge of studying it. Wiltsey Stirman and colleagues [3] suggested several reasons for this challenge. First, practices frequently fail to adapt to changes in context and end up discontinuing, limiting the sample size of sites to be studied for sustainment. Second, sustainment often is defined in different ways across studies, which in turn influences the manner that it is assessed. Other studies also suggest that sustainment may not be assessed in implementation studies if the focus of the study is on the initial uptake by early adopters [9]. While studies in recent years have examined sustainment rates, most pertain to specific programs or practices, which limits the generalizability of such results. For example, a study by Ford et al. examined the sustainment of the process improvement model NIATx200, finding that between 26.7 and 40.8% of clinics sustained at least one of the three initiated improvements 2 years after starting up, while 11.6% of clinics sustained for two of three improvements and 5% may have sustained all three [10]. Other studies may evaluate multiple evidence-based programs (EBPs) but differ on the levels of analysis: Brookman-Frazee et al. examined sustainment on a therapist level within the Los Angeles County Mental Health Context, finding that the average length of time between the first and final claims for reimbursement (for the delivery of any EBP) was 21.71 months (with a standard deviation of 16.32 months) [11]; Bond et al. examined sustainment on the site level, finding that 79.6% of sites in the National Implementing Evidence-Based Practices Project were sustained for 4 years following start-up, while 47% sustained 8 years following start-up [12]. Other evaluations relied on qualitative data alone [13] and unreliable self-reported data [14, 15] or were unable to examine fidelity altogether [16, 17].

A number of reviews have also examined rates of sustainment. A review of evidence-based health and family studies conducted in disadvantaged communities found that only 43% of studies reported sustaining at least half of their participating sites for 2 or more years following the point of provider training [18]. Within its sample of studies, less than half of the articles defined sustainment, and nearly all of these definitions referred only to the continuation of the practice, project, or activity without indication of time. Wiltsey Stirman and colleagues’ systematic review [3] attempted to define a generalized rate of sustainment, separating out studies by the level of analysis (e.g., patient, site, provider), and found that of the 4 studies that had independent reporting of provider-level sustainability/fidelity outcomes, fewer than half of the providers achieved “high” sustainment in terms of skill, intensity, and fidelity. Most studies used the completion of initial implementation as the starting point for the sustainability timeframe, but these reviews illustrate broader definitional issues with sustainment: they might have different definitions of the completion of implementation (following provider training versus the end of grant funding) and differ on the level of analysis (proportion of studies reporting that a majority of sites sustained versus proportion of EBPs sustained).

Wiltsey Stirman et al. [3] also noted that their attempts to define a sustainment rate were limited by the variability in the rigor of evaluation used by the studies reviewed. For instance, most of the studies were conducted retrospectively [3]. A generalized sustainment rate would be useful in setting realistic expectations for purveyors, organizational leadership, and policymakers regarding the likelihood of sustainment of programs in the community, and as a target that future implementations can aim to exceed.

Discovering a generalized rate of sustainment requires a dataset of real-world implementation efforts with a sufficiently rigorous methodology and sample size large enough to overcome the previously noted barriers. One unique opportunity to examine the rates of sustainment from a wide range of real-world implementations is the Universal Stages of Implementation Completion [19, 20], derived from the Stages of Implementation Completion (SIC) [21].

### The Universal Stages of Implementation Completion

**Fig. 1:**
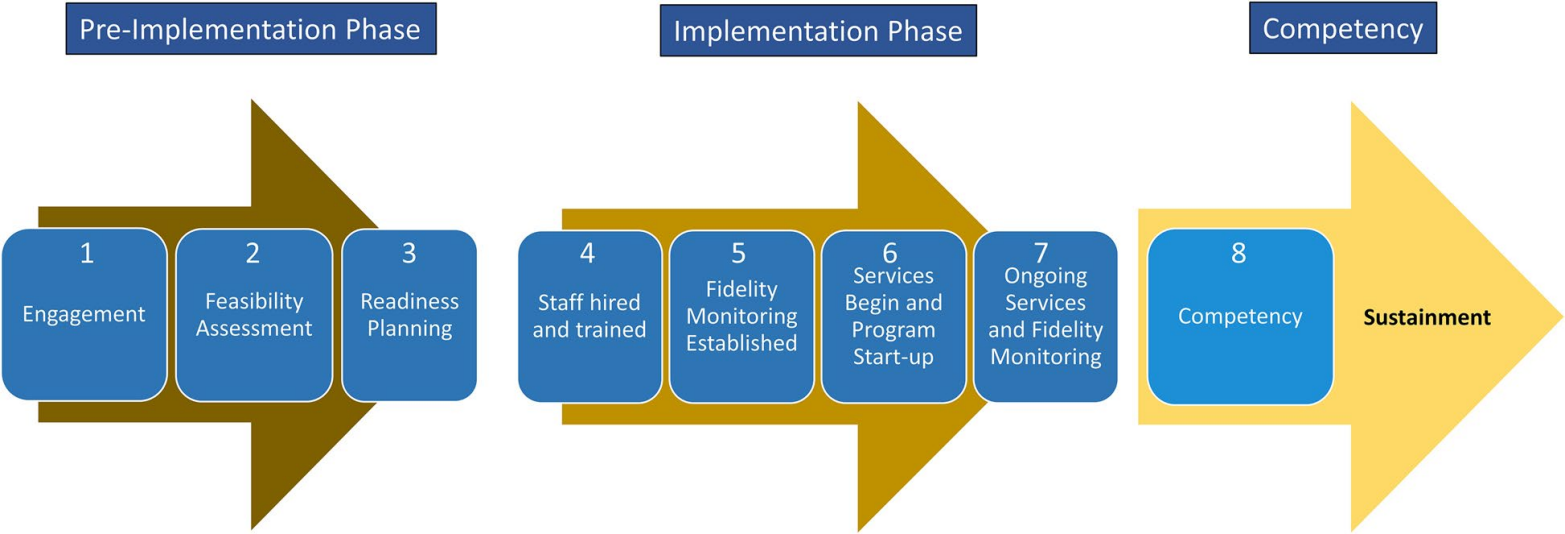
Infographic of the Stages of Implementation Completion (SIC)

The SIC is a standardized tool to track the implementation process and milestone completion across different phases: pre-implementation, implementation, and sustainment [22]. Initially created for an implementation trial of Treatment Foster Care Oregon (TFCO; formerly known as Multidimensional Treatment Foster Care) [23], the SIC measures completion of activities in the implementation process along eight distinct stages. This tool has empirical support for predicting variations in implementation success. For instance, a study regarding predictive validity of the SIC for TFCO found that implementation behavior—defined by proportion and duration of activities completed—in the first three stages (i.e., pre-implementation) predicted the likelihood of reaching stage 6: the point at which the first client is served a.k.a. “program start-up” [24]. Figure 1 displays the eight SIC stages building toward sustainment.

To evaluate the applicability of the SIC in measuring other EBPs, a process of *adaptation* was undertaken [22]. SIC adaptation methods include the SIC research team sitting and working with developers of EBPs to operationalize the process of an ideal implementation for each respective EBP [25]. Although successful in developing a consistently valid and reliable measurement framework for implementation processes across EBPs, this adaptation process was individualized for each given practice and was a time- and resource-intensive undertaking. Across numerous adaptations, the SIC research team came to identify implementation activities that were common across them [19]. These common, or “universally used implementation activities,” were used to develop the Universal SIC (UniSIC)—a standardized measure of the implementation process.

The UniSIC is intended to serve as the base for standardized measurement of the implementation process of a wide range of EBPs, such as those implemented in justice systems [26], schools [27], and public health [28]. It has demonstrated strong psychometric properties and has been used by 34 practices to measure implementation processes and milestones.

The UniSIC allows for comparisons between implementations by different practices. In addition to the original EBPs, many practices still select to adapt individually tailored SICs; however, because the UniSIC includes activities most commonly expected during implementations, UniSIC items still are included in the adapted SICs, allowing for inclusion of their data in cross-practice analyses. Thus, data are available on common implementation processes and milestones across the majority of practices that have collected data with the SIC or UniSIC, providing a unique opportunity to examine sustainment in a relatively large sample of implementation efforts measured on a common scale.

The SIC conceptualizes sustainment as a post-implementation stage (involving the continuation of service delivery), albeit with the potential for identifying sustainment as an implementation outcome. This post-implementation stage begins after the site being tracked is certified as competent in the model by the purveyor of the practice. The SIC thus largely conceptualizes sustainment through the *linear *view rather than the *dynamic* view [29]; the linear view identifies sustainment as the final stage of implementation and emphasizes the maintenance of intervention fidelity as a crucial element of sustainment.

### Current study

Few, if any, studies have examined rates of sustainment in a large dataset involving organizations from multiple service sectors. Leveraging the UniSIC data available across a range of real-world practices, this study aims to conduct descriptive analyses to begin building hypotheses about implementation processes that help support sustainment. The proportion of sites are identified that (a) achieved competence in program delivery thus having the opportunity to sustain and (b) sustained competent programs under two different definitions of sustainment.

## Methods

### Sample

Data for the current sample come from a larger repository of SIC data, collected from a range of practices implementing in different service sectors. Of the 1778 sites in the SIC database at the time of data capture, 60% were in the USA, 7% were international (e.g., Canada, Australia, Mozambique, Denmark, etc.), and the remainder were missing region identification. As part of the SIC user agreement, practices agree to allow the use of their implementation data for ongoing analyses in a de-identified way. All data are entered directly into the secure SIC online data entry tool by the purveyors of participating practices. All data are validated and scored using standardized and automated protocols that were programmed into the Web-based platform for access by users at any time (https://sic.oslc.org/SIC). Table 1 displays sample activities that are recorded in the SIC.

**Table 1:** Examples of stage activities in the UniSIC

Stages	Activities
Stage 1	Engagement
1.02	Date of interest indicated
1.03	Date agreed to consider implementation
Stage 2	Feasibility
2.01	Date of first site planning contact
2.03	Date that the Feasibility Questionnaire is completed
Stage 3	Readiness planning
3.03	Date of recruitment review
3.07	Date of written implementation plan completed
Stage 4	Staff hired and trained
4.01	Date that first clinical staff is hired
4.02	Date that the program supervisor is trained
Stage 5	Fidelity monitoring
5.01	Date that fidelity system training is held
5.04	Date that the IT technician is identified
Stage 6	Services and intervention/services consultation begin
6.01	Date of the first client served
6.03	Date of the first clinical team and/or supervision meeting fidelity review
Stage 7	Ongoing service delivery and fidelity monitoring
7.01	Date of the first site visit
7.07	Date of program fidelity assessment
7.11	Date that key supervision activities meet the fidelity threshold
Stage 8	Competency
8.02	Date that site is certified competent
8.04	Date first supervisor certified

The UniSIC dataset includes demographic data for each site, as well as stage- and phase-specific data on individual activities. The UniSIC dataset is “live” and so includes sites that were active at the time of analysis. The dataset used for the current analysis was captured from the SIC repository on September 27, 2020.

Of the total 1778 sites that were represented at the time of data capture, the sample was filtered to sites that had achieved program start-up—defined as when a site served its first client—limiting the sample size to *N* = 522 sites. Of these, 209 sites were actively engaging in ongoing implementation activities and were excluded from analysis (thus maintaining the future opportunity to achieve sustainment), leaving a sample of sites to evaluate regarding achievement of competency in service delivery (i.e., Competency Available Sample; *N* = 313). To arrive at the sample of sustained sites, additional sites were excluded—105 sites did not have a start-up date or competency date or did not have follow-up data after achieving competency; these data points were necessary for sustainment analysis. The Sustainment Available Sample therefore included *N* = 208 sites.

**Fig. 2:**
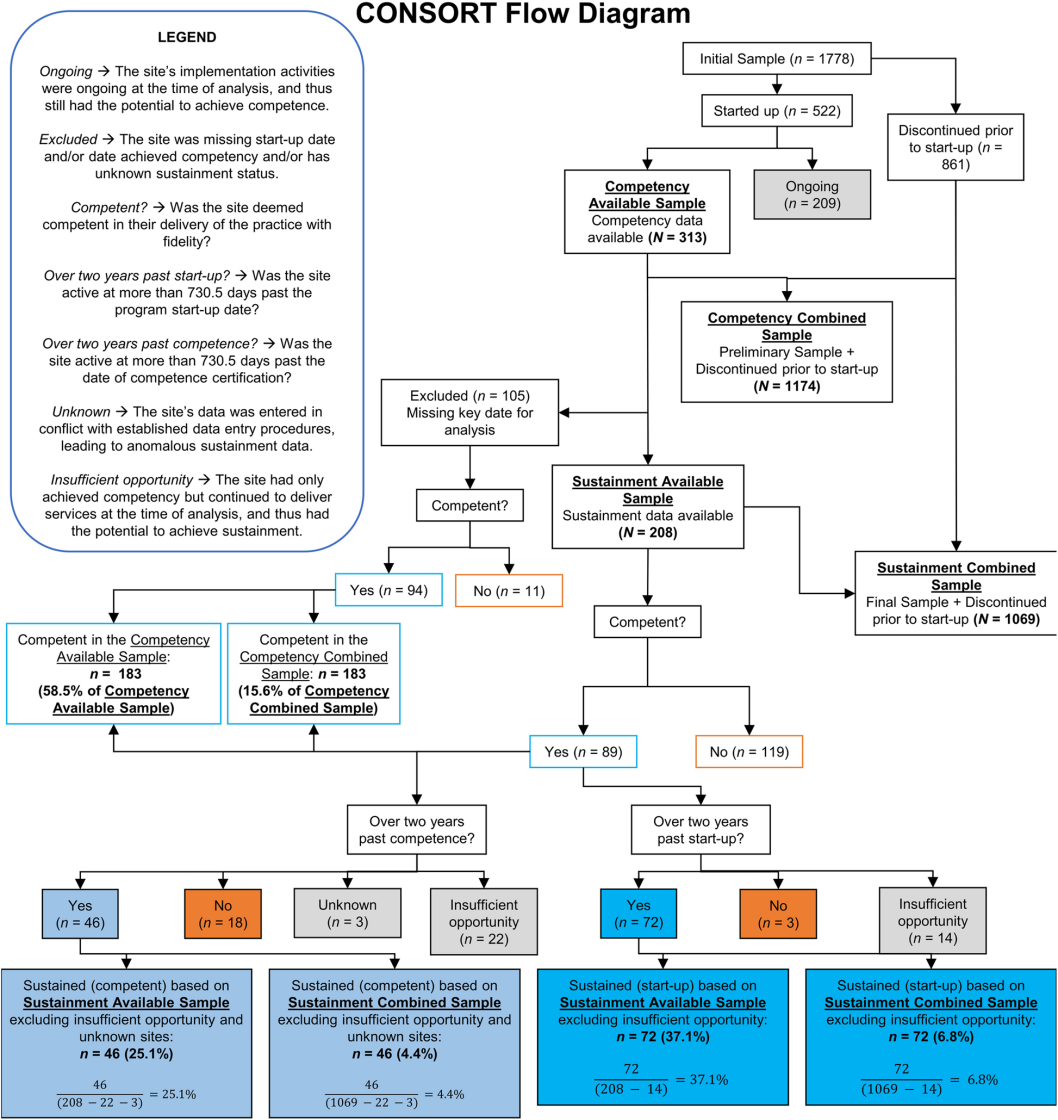
The CONSORT diagram of sites implementing an evidence-based practice showing rates of achieving (1) competency, (2) 2-year sustainment post-program start-up, and (3) 2-year sustainment post-competency

Considering the value of finding a rate of sustainment based on a sample that includes the larger set of sites that did not start-up (i.e., an “overall” rate, not merely a rate for sites that had advanced far enough in the implementation to start up a program), sites that discontinued prior to start-up were added to the Competency Available Sample and Sustainment Available Sample to form the Competency Combined Sample and Sustainment Combined Sample, respectively. There were 861 sites that had been discontinued prior to start-up; hence, the Competency Combined Sample had 1174 sites, while the Sustainment Combined Sample had 1069 sites. The number of sites found to fulfill the criteria for competence and for each definition of sustainment has the respective combined samples as the denominators to find each rate. The CONSORT diagram (Fig. 2) details these subdivisions and sample sizes. The proportions of sites by region remain generally consistent when the samples are parsed accordingly (e.g., in the Sustainment Combined Sample, 78% are listed in the USA, 7% are listed as international, and the remainder were missing region identification).

### Data analysis

Analyses were conducted in the R statistical environment (R version 3.6.1) [30], using the tidyverse [31], lubridate [32], and readxl [33] packages. Analyses were also replicated in SPSS (version 26.0.0.1).

### Defining sustainment

The recommendations by Wiltsey Stirman and colleagues inform the definitions of sustainment. They recommend that sustainment of sites be measured “two or more years after implementation” (p.11) [3]. However, it is unclear from where along the continuum of implementation activities the 2 years is counted. Some starting points include the initiation of program training as a starting date for measuring sustainment [18] or the initiation of service delivery as a starting date [12]. Given that the intervening period between program training and service delivery may be substantial (in the Sustainment Available Sample, a median of 64 days and a range of 0 to 620 days were found) and that the emphasis of this paper’s definition of sustainment is service delivery, the initiation of service delivery might be a more meaningful starting point. An alternative is suggested by the definition of sustainment within the RE-AIM framework; it defines implementation as “the extent to which a program is delivered as *intended* [emphasis added]” (p.1323) [34]. Thus, the starting point from which sustainment should be measured would be when services are delivered *competently* (i.e., SIC stage 8; see Fig. 1). This is consistent with the SIC process model (Fig. 1) since it subscribes to the linear conception of sustainment.

Data from the UniSIC were used to construct two definitions of sustainment. Included UniSIC variables were the dates of (1) program start-up, (2) competence and/or certification, (3) last recorded implementation activity completed, and (4) an indicator variable recording whether a program had been certified competent at any point during its lifespan. These data points permitted the analysis of two overlapping definitions of sustainment. The first defines a site as having sustained if it has been certified competent and continues to deliver services for at least 2 years (730.5 days) past the program start-up date, henceforth referred to as “sustained (start-up).” The second definition of sustainment also requires that a site is certified competent but differs in requiring that the site continue to deliver services for at least 2 years past the date of competence and/or certification, henceforth referred to as “sustained (competent).” Both definitions are reflected in the results shown in Table 4 and Fig. 2.

## Results

### Represented service sectors

Sites included in the Competency Combined Sample and Sustainment Combined Sample are reflected in Table 2, while sites included in the Competency Available Sample and the Sustainment Available Sample are reflected in Table 3. Of note, 10 service sectors in the SIC repository were represented in the Sustainment Combined Sample and 7 service sectors in the SIC repository were represented in the Sustainment Available Sample.

**Table 2:** Sites by service sector (Competency Combined/Sustainment Combined Sample)

Service sector	Competency Combined Sample		Sustainment Combined Sample	
	*n*	*n*	*n*	%
Child welfare	184	15.7	178	16.7
Public health	5	0.4	5	0.4
DHS prevention	3	0.3	2	0.2
Criminal justice	11	0.9	9	0.8
Education/school	160	13.6	145	13.6
Substance use	167	14.2	113	10.6
Primary care	110	9.4	102	9.5
Mental health care	4	0.4	3	0.3
Community-based	2	0.2	2	0.2
Juvenile justice	119	10.1	118	11.0
Missing	409	34.8	392	36.7
Total	1174	100.0	1069	100.0

**Table 3:** Sites by service sector (Competency Available/Sustainment Available Sample)

Service sector	Competency Available Sample		Sustainment Available Sample	
	*n*	%	*n*	%
Child welfare	30	9.6	24	11.5
DHS prevention	2	0.6	1	0.5
Criminal justice	6	1.9	4	1.9
Education/school	26	8.3	11	5.3
Substance use	107	34.2	53	25.5
Primary care	18	5.8	10	4.8
Mental health care	1	0.3	0	0.0
Juvenile justice	7	2.2	6	2.9
Missing	116	37.1	99	47.6
Total	313	100.0	208	100.0

### Rates of competence/sustainment achievement

As seen in the CONSORT diagram (Fig. 2), sites in the original sample were divided into several subcategories: a Competency Available Sample (*N* = 313), which included sites for which competency data was available, and a Sustainment Available Sample (*N* = 208), in which sustainment achievement could be evaluated. Ongoing sites (*n* = 209) were excluded from the analysis. Figure 2 displays the rates of competency and sustainment found in the respective samples. In the Competency Available Sample, 183 sites (58.5% of the Competency Available Sample) achieved competency. In the Sustainment Available Sample, the rates of sustainment were found to differ greatly when defined with reference to the program start-up date (*n* = 72; 37.1% of the Sustainment Available Sample) versus to the date that the program was first delivered with competency (*n* = 46; 25.1% of the Sustainment Available Sample). Given the ongoing sites and those that are competent but have had insufficient opportunity to achieve sustainment definitions (*n* = 14 for sustained (start-up) and *n* = 22 for sustained (competent), respectively), the sustainment rate may change if these sites either fail to or go on to fulfill the criteria for sustainment.

When these sites are considered under the larger Competency Combined Sample (*N* = 1174) and Sustainment Combined Sample (*N* = 1069), respectively, the rate of competence is 15.6%, the rate of sustained (start-up) is 6.8%, and the rate of sustained (competent) is 4.4%. These rates represent the “overall” rate from all implementation attempts tracked with the SIC, as opposed to limiting the rate among sites that at least started up a program.

Descriptive proportions of the sites across the four distinct samples (Competency Combined Sample, Sustainment Combined Sample, Competency Available Sample, Sustainment Available Sample) can be found in Table 4. Note that the proportions and percentages in the table may not reflect the actual rates of sustainment, given that the sites in the categories of *insufficient opportunity* and *unknown* are excluded from the denominators of the rates.

**Table 4:** Proportions and percentages of sites that had achieved competence/sustainment across the Competency Combined, Sustainment Combined, Competency Available, and Sustainment Available Samples

Competency Combined Sample		Total	
		*n*	%
Competent			
Yes		183	15.6
No		991	84.4
Total		1174	100.0
Sustainment Combined Sample		Total	
		*n*	%
Sustained (start-up)^a^			
Yes		72	6.7
No		983	91.2
Insufficient opportunity		14	1.3
Sustained (competent)^b^			
Yes		46	4.3
No		998	93.4
Insufficient opportunity		22	2.1
Unknown		3	0.3
Total		1069	100.0
Competency Available Sample		Total	
		*n*	%
Competent			
Yes		183	58.5
No		130	41.5
Total		313	100.0
Sustainment Available Sample		Total	
		*n*	%
Sustained (start-up)^a^			
Yes		72	34.6
No		122	58.7
Insufficient opportunity		14	6.7
Sustained (competent)^b^			
Yes		46	22.1
No		137	65.9
Insufficient opportunity		22	10.6
Unknown		3	1.4
Total		208	100.0

^a^The site has been certified competent and has remained active for at least 2 years (730.5 days) past the *date that a program at a site starts up*

^b^The site has been certified competent and has remained active for at least 2 years (730.5 days) past the *date of competence and/or certification achieved*

## Discussion

The rates of competency and sustainment found among sites that started up were substantially higher than the rates of competency and sustainment found when including all sites. Although the definition for the achievement of site competency is defined by the purveyor of each practice, reaching this milestone indicates that the purveyor of the practice has verified the site as delivering services to an acceptable degree of fidelity. Measures of fidelity differ from practice to practice, but typically include some observation of program delivery and functioning.

### Rates of competence

The rate of competence among the sites that started up (in the Competency Available Sample) was 58.5%; however, this rate fell to 15.6% when taken as a proportion of the Competency Combined Sample. Given that competence serves as an indicator of fidelity to the intervention, which is thought to be an important feature of sustainment [30], it is sobering to recognize that even among sites that started up, less than six out of 10 sites achieved it. Achieving start-up of the EBP is indicative of a high level of commitment among sites — many of these sites will have invested a substantial amount of resources toward conducting pre-implementation activities, building implementation support systems, and conducting training [35] — yet a substantial proportion of these sites did not go on to achieve competence.

### Rates of sustainment

Depending on the choice of definition, approximately 25.1% or 37.1% of sites that started up achieved sustainment. This rate may differ from the true rate of sustainment, given that this rate is limited to estimates from sites that were confirmed to have achieved sustainment at the time of analysis. Active sites (*n* = 209) still had the potential for achieving competence and/or sustainment. Although they were excluded from analyses, these sites may yet achieve sustainment by either definition if they remain active; they also could discontinue, and in either case, the denominator would simultaneously increase. Notably, sites that continue to deliver services for at least 2 years past start-up but have *not* been certified competent (comprising 44 of the 208 sites (21.2%) in the Sustainment Available Sample) were also not contained in either category of sustained sites. While active sites within this category may go on to be certified as competent, the category was ultimately excluded because they are struggling to deliver the program well enough to start the sustainment clock.

Based on the combined samples, the rate of sustained (start-up) is 6.8% and the rate of sustained (competent) is 4.4%. This implies that less than one in 10 sites that seek to implement a practice go on to sustain the practice for 2 years past initiating services and/or achieving competence in delivering services within the practice. Similar to the rate based on sites that started up, this rate also excluded sites that remain active, and they may go on to achieve sustainment or may discontinue. It is notable that many of the sites in the combined samples discontinued early — 826 out of the 861 sites (95.9%) that discontinued prior to start-up did so in the pre-implementation phase (SIC stages 1, 2, and 3; see Fig. 1). This suggests that many of the implementation “failures” occurred very early on in the implementation process and may speak to a trend of discontinuation before fully committing to implementation efforts (e.g., before clinical staff are hired, or before program training is initiated). This also highlights an opportunity to increase adoption and sustainment rates by improving strategies to support sites through pre-implementation.

The reduction in rates of sustainment when measured from program start-up versus competency and/or certification are not surprising. The sustainment literature aligned with the linear view of sustainment suggests that the rate of sustainment of EBPs tends to drop off over time. For instance, Bond et al. [12] found in their study of sustainment among psychosocial EBPs in routine mental healthcare settings that 79.6% of sites continued 4 years after starting up, but this rate dropped to 47% 6 years after starting up [26].

### Strengths of the study

The UniSIC data reflect multiple realities that lend themselves to an accurate estimate of the rate of sustainment among community-based practices. First, this study is not affected by publication bias, which may have impacted the studies included in previous systematic reviews. Publication bias is the phenomenon that the results of published studies systematically differ from unpublished studies, often because studies that find successful outcomes are more likely to be published by journals [36]. In the case of the systematic reviews from which this paper is drawing comparisons, the reviewed studies might have been more likely to be published, and therefore included in the review, if the outcomes indicated successful sustainment. Health services research, which encompasses much of the sustainment literature that was reviewed, is thought to have strong incentives to publish results indicating beneficial outcomes, though the extent to which publication bias affects the field is unclear [37]. For this reason, such reviews are likely to overestimate the true rate of sustainment. Similarly, by definition, systematic reviews are based on published findings, often of grant-funded research where research dollars are used to develop sites, which provide resource support that may contribute to more successful outcomes. In contrast, the UniSIC data includes both successful and unsuccessful implementation and sustainment attempts, from implementations occurring under a range of rigor and funding sources in real-world settings.

A second advantage of this study is that our sustained (competent) definition incorporates competence into the definition of sustainment. Wiltsey Stirman et al. recommend that definitions of sustainment should incorporate concepts related to fidelity (e.g., maintenance of program core elements) [3]. By incorporating competence and/or certification into our definition, it allows us to ascertain that the sites in question have previously been certified as competent during the sustainment period, which explicitly includes delivery of services with fidelity. This falls short of measuring fidelity at the 2-year point past the competence and/or certification date — the UniSIC does not measure the fidelity and competence of sites at follow-up — but our definition marks an improvement over other definitions that fail to incorporate fidelity.

Third, to the authors’ knowledge, this study is the first to utilize a large and diverse sample of sites and practices to identify a sustainment rate. Although a majority of the studies included in the review by Wiltsey Stirman and colleagues examined sustainment in multiple sites and settings, none of the samples studied included programs across multiple domains (e.g., healthcare, education, child welfare, mental healthcare) [3]. As seen in Tables 2 and 3, the greatest proportion of sites in this sample were in the substance use, education, primary care, juvenile justice, and child welfare service sectors. Although there was a large proportion of missing data for service sectors, the sustainment rate found could provide a reference for the types of practices represented in the sample.

A fourth advantage of this study is that it addresses one of the limitations noted by Wiltsey Stirman and colleagues [3]. The studies that they reviewed had varying levels of rigor of evaluation limiting finding a generalizable rate of sustainment, whereas this study employs the same rigor of evaluation across the sample.

### Limitations

Several methodological limitations are noteworthy. First, a small but noteworthy proportion of sites in our original sample (*n* = 105) were excluded from our sustainment sample because they either lacked a program start-up and/or competency date or lacked follow-up data. This could be addressed in future research by retrospectively collecting the missing data. Second, although there is a high degree of confidence that the entered data accurately reflects the state of the sites, purveyors might not immediately enter data as soon as they become aware of it, impacting the accuracy of analyses. Future research on sustainment in the UniSIC could develop methods to directly contact sites to update the SIC database regarding long-term sustainment. Third, sites which are represented in the SIC database might be more likely to sustain than sites adopting programs that do not use the SIC. That is, purveyors of EBPs that use the SIC measurement tool to support their implementation efforts might offer a more rigorous quality of technical assistance than other program purveyors. Thus, an indirect selection effect might exist with sites using the UniSIC adopting EBPs that are more likely to achieve successful implementation and sustainment because of a belief in tracking the implementation process and outcomes, which may then improve their likelihood of high-quality implementation.

### Future directions

Overall, the UniSIC estimate can serve as a useful comparison for future research on sustainment and provide realistic expectations for key implementation stakeholders regarding the likelihood of sustainment of programs in the community. Future researchers of sustainment should look to previous work on sustainment to construct their definition, explicitly stating inclusion and exclusion criteria in the sample, and choosing an appropriate start- and end-point to determine sustainment outcomes.

The UniSIC benefits from its well-validated measurement of the implementation process and its ease of use by implementation researchers to track the process. The UniSIC also might have utility as a guide to facilitate successful implementation, given its ability to predict success for program start-up [24]. Indeed, a current randomized trial is evaluating the impact of including SIC data as a part of the purveyor feedback to sites, with promising preliminary outcomes (R01 DA044745). The large number of practices and sites being tracked by the UniSIC make it a valuable repository for analyses regarding common implementation activities across a wide variety of practices. As the pool of data from the UniSIC continues to grow, future implementation research should seek to track sustainment rates.

A more comprehensive assessment of sustainment would require the measurement at several points in time, typically in the years following competence and/or certification and involve the assessment of sustainment using measures of fidelity (while also accounting for potentially valid adaptations) [2, 3]. Funding mechanisms should be made available that consider the time needed for the assessment of sustainment to occur, recognizing the need for tracking more than 2 years past the point of competence.

The SIC dataset captures the proportion and duration of stages and phases for each site. An important next step for this line of sustainment research is to capture how these proportions and durations predict site sustainment; similar research previously has been conducted regarding the prediction of site start-up [24]. Such a predictive model could be useful to future practices utilizing the SIC; feedback regarding their likelihood of sustainment could motivate purveyor and organizational efforts to support the implementation attempt, improving the likelihood of sustainment.

The SIC dataset is an active dataset that continues to track the implementation processes of sites. It is noteworthy that our data download was conducted in September 2020— approximately 6 months into the COVID-19 pandemic. Out of the 208 sites in the Sustainment Available Sample, 19 sites that achieved the sustained (start-up) status de-adopted between March 2020 (the approximate onset of pandemic disruptions in the USA) and the time of capture of the data. A recently funded COVID-19 supplement supported the development of a SIC enhancement that now allows for assessment of outer context impacts, such as COVID-19 disruptions, on the implementation process. Future analyses will allow for closer examination of the impact of outer context factors on the implementation and sustainment of EBPs.

## Conclusions

The estimated rates of competence and sustainment among the sites that started up were 58.5% for competence, 37.1% for sustained (start-up), and 25.1% for sustained (competent). The rates of competence and sustainment among the combined samples were far lower: 15.6% for competence, 6.8% for sustained (start-up), and 4.4% for sustained (competent). These rates may help to set realistic expectations for purveyors, organizational leadership, and policymakers regarding the likelihood of sustainment of programs in the community, and as a target that future implementations can aim to exceed. Certain relevant characteristics of the dataset from which this rate is drawn suggest that the rates found may be accurate to the true rates of sustainment among programs in the community, and possibly among programs in general. Future research on rates of sustainment should carefully define their measures of sustainment and seek to center their analyses on implementation under real-world conditions. Innovative tools such as the UniSIC might provide standardized strategies for moving our understanding of sustainment significantly forward, creating opportunities that are better informed to improve the implementation of EBPs.

## Data Availability

The datasets used and/or analyzed during the current study are stored in a password-protected, secure repository of like data and are available from the corresponding author on reasonable request.

## Abbreviations

EBP: Evidence-based practice
RE-AIM: Reach, effectiveness, adoption, implementation, and maintenance
SIC: Stages of Implementation Completion
UniSIC: Universal Stages of Implementation Completion
